# Effectiveness of AI for Enhancing Computed Tomography Image Quality and Radiation Protection in Radiology: Systematic Review and Meta-Analysis

**DOI:** 10.2196/66622

**Published:** 2025-02-27

**Authors:** Subo Zhang, Zhitao Zhu, Zhenfei Yu, Haifeng Sun, Yi Sun, Hai Huang, Lei Xu, Jinxin Wan

**Affiliations:** 1 Department of Medical Imaging The Second People's Hospital of Lianyungang Lianyungang China; 2 Department of Medical Imaging Cancer Hospital of Lianyungang Lianyungang China; 3 Lianyungang Clinical College Jiangsu University Lianyungang China

**Keywords:** artificial intelligence, computed tomography, image quality, radiation protection, meta-analysis

## Abstract

**Background:**

Artificial intelligence (AI) presents a promising approach to balancing high image quality with reduced radiation exposure in computed tomography (CT) imaging.

**Objective:**

This meta-analysis evaluates the effectiveness of AI in enhancing CT image quality and lowering radiation doses.

**Methods:**

A thorough literature search was performed across several databases, including PubMed, Embase, Web of Science, Science Direct, and Cochrane Library, with the final update in 2024. We included studies that compared AI-based interventions to conventional CT techniques. The quality of these studies was assessed using the Newcastle-Ottawa Scale. Random effect models were used to pool results, and heterogeneity was measured using the *I*² statistic. Primary outcomes included image quality, CT dose index, and diagnostic accuracy.

**Results:**

This meta-analysis incorporated 5 clinical validation studies published between 2022 and 2024, totaling 929 participants. Results indicated that AI-based interventions significantly improved image quality (mean difference 0.70, 95% CI 0.43-0.96; *P*<.001) and showed a positive trend in reducing the CT dose index, though not statistically significant (mean difference 0.47, 95% CI –0.21 to 1.15; *P*=.18). AI also enhanced image analysis efficiency (odds ratio 1.57, 95% CI 1.08-2.27; *P*=.02) and demonstrated high accuracy and sensitivity in detecting intracranial aneurysms, with low-dose CT using AI reconstruction showing noninferiority for liver lesion detection.

**Conclusions:**

The findings suggest that AI-based interventions can significantly enhance CT imaging practices by improving image quality and potentially reducing radiation doses, which may lead to better diagnostic accuracy and patient safety. However, these results should be interpreted with caution due to the limited number of studies and the variability in AI algorithms. Further research is needed to clarify AI’s impact on radiation reduction and to establish clinical standards.

## Introduction

Computed tomography (CT) has revolutionized medical imaging since its introduction, providing high-resolution, 3D visualizations of anatomical structures. Its widespread adoption has significantly enhanced diagnostic capabilities across various medical specialties [1-3]. However, the increasing use of CT scans has raised concerns about radiation exposure and its potential long-term health effects on patients [4,5].

Simultaneously, maintaining optimal image quality is crucial for accurate diagnosis and treatment planning. This creates a challenging balance between minimizing radiation dose and preserving diagnostic image quality [6-8]. In recent years, artificial intelligence (AI) has emerged as a promising solution to address the balance between maintaining high image quality and minimizing radiation dose in CT imaging [9-11].

AI, particularly deep learning algorithms, has shown potential in various aspects of CT imaging, including image reconstruction, noise reduction, and automated image analysis [12]. These AI-driven approaches aim to enhance image quality while allowing for lower radiation doses, potentially improving both diagnostic accuracy and patient safety [13].

Several studies have investigated the application of AI in CT imaging, focusing on different anatomical regions and clinical scenarios. For instance, Hu et al [14] explored the use of deep learning models for intracranial aneurysm detection on CT angiography images. Zhang et al [15] investigated AI reconstruction algorithms in CT imaging of sports injuries. Other researchers have examined AI’s role in breast cancer radiotherapy planning [16] and liver CT imaging [17].

Despite the growing body of research, the overall effectiveness of AI in improving CT image quality and reducing radiation dose remains unclear [14,17]. The variability in study designs, AI algorithms, and outcome measures makes it challenging to draw definitive conclusions about the broader impact of AI on CT imaging practices [18]. This meta-analysis aims to synthesize the available evidence on the role of AI in CT image quality control and radiation protection, providing a comprehensive evaluation of AI’s role across various clinical scenarios and anatomical regions. Our study contributes to the literature by offering a systematic overview of the current state of AI in CT imaging, which can inform future research directions and clinical applications.

## Methods

### Search Strategy and Study Selection

A comprehensive literature search was conducted across multiple electronic databases, including PubMed, Embase, Web of Science, Science Direct, and Cochrane Library. The search strategy used a combination of Medical Subject Headings terms and keywords related to AI, deep learning, and CT imaging. The specific search string used was as follows: (Intelligence, Artificial [Title/Abstract]) OR (Computer Reasoning [Title/Abstract]) OR (Reasoning, Computer [Title/Abstract]) OR (AI (Artificial Intelligence [Title/Abstract]) OR (Machine Intelligence [Title/Abstract])) and so on (Multimedia Appendix 1).

The selected databases are renowned for their extensive coverage of medical and scientific literature, ensuring a thorough retrieval of studies pertinent to our research question. The choice of these databases was also influenced by their indexing of a wide array of medical journals and conference proceedings, which are critical for capturing the latest advancements in the field of AI and CT imaging.

The search was conducted without language restrictions and conducted from the inception of the databases until the search date in 2024, to ensure inclusion of the most current and relevant studies.

### Inclusion and Exclusion Criteria

Studies were eligible for inclusion if they met the following criteria: (1) clinical validation studies comparing AI-based interventions with conventional CT imaging techniques; (2) reported outcomes related to image quality, radiation dose, or diagnostic performance; and (3) provided sufficient data for quantitative analysis. Exclusion criteria included (1) nonclinical studies or (2) those without a control group, (3) studies focusing solely on AI development without clinical validation, (4) review articles, (5) case reports, (6) conference abstracts, and (7) studies with insufficient data for meta-analysis.

### Data Extraction and Quality Assessment

Two independent reviewers extracted data from the included studies using a standardized form. The extracted information included study characteristics (author, year, and study design), sample size and patient demographics, AI intervention details, and outcome measures (image quality metrics, radiation dose indicators, and diagnostic performance).

The quality of the included studies was assessed using the Newcastle-Ottawa Scale for nonrandomized studies. This scale evaluates studies based on selection, comparability, and outcome domains, with a maximum score of 9 indicating the highest quality. The risk of bias for each included study was assessed using the Cochrane risk of bias tool, which provides a standardized evaluation of study quality.

### Statistical Analysis

Meta-analyses were performed using Review Manager (version 5.4; The Cochrane Collaboration). For continuous outcomes (eg, image quality scores and CT dose index), mean differences with 95% CIs were calculated. For binary outcomes (eg, diagnostic accuracy), odds ratios with 95% CIs were computed.

Random effect models were used to account for potential heterogeneity among studies. Heterogeneity was assessed using the *I*² statistic, with values of 25%, 50%, and 75% considered as low, moderate, and high heterogeneity, respectively.

Publication bias was assessed visually using funnel plots. Sensitivity analyses were conducted by sequentially removing each study to evaluate its impact on the overall effect size.

Results were considered statistically significant at *P*<.05. Forest plots were generated to visually represent the meta-analysis results, displaying individual study effects and the pooled effect size.

### Ethical Considerations

An ethical statement is not applicable because this study is based exclusively on published literature.

## Results

### Study Selection and Characteristics

**Figure 1:**
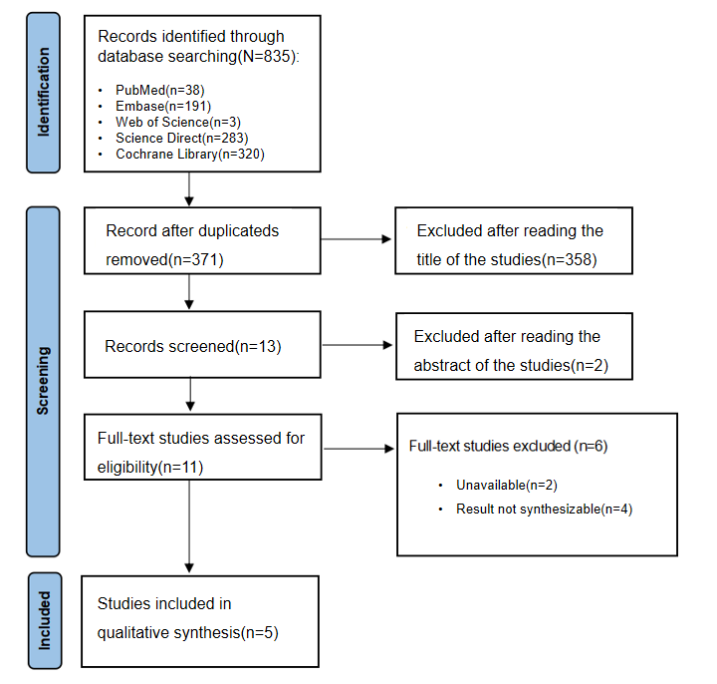
Preferred Reporting Items for Systematic Reviews and Meta-Analyses (PRISMA) flow diagram for study selection, detailing the process from initial records to final inclusion across various databases and time frames.

The initial database search yielded 835 records (PubMed: n=38, Embase: n=191, Web of Science: n=3, Science Direct: n=283, and Cochrane Library: n=320). After removing duplicates, 371 unique records remained. Following title and abstract screening, 11 full-text articles were assessed for eligibility. Ultimately, 5 studies met the inclusion criteria and were included in the meta-analysis (Figure 1).

The 5 included studies were all clinical validation studies published between 2022 and 2024. They encompassed a total of 929 participants (464 in the experimental AI group and 465 in the control group). The studies focused on various anatomical regions and clinical applications, including intracranial aneurysm detection [14], knee anterior cruciate ligament injury [15], breast cancer radiotherapy planning [16], head CT angiography [9], and liver CT imaging [17].

All included studies used deep learning–based AI algorithms for image reconstruction or analysis. The sample sizes ranged from 60 to 14,715 participants. The Newcastle-Ottawa Scale scores for the included studies ranged from 7 to 8, indicating good methodological quality (Table 1).

**Table 1:** Basic characteristics of included literature.

Author (year)	Type of invention group	Type of study	Training dataset, n	Internal validation dataset, n	External validation dataset, n	Exposure factors	NOS^a^ score
Hu et al [14] (2024)	AI^b^	Clinical validation study	12,817	1700	1198	Accuracy, sensitivity, positive predictive value, and negative predictive value	7
Zhang et al [15] (2022)	AI	Clinical validation study	30	30	30	Image quality, CTDI^c^ volume, and dose length generation (DLP^d^)	_8_
Ma et al [16] (2023)	AI	Clinical validation study	230	280	22	Image quality	8
Huang et al [9] (2023)	AI	Clinical validation study	63	38	25	CTDI, CM^e^ dose, and image quality	7
Lee et al [17] (2024)	AI	Clinical validation study	296	246	90	CT image noise level and diagnostic performance	_8_

^a^NOS: Newcastle-Ottawa Scale.

^b^AI: artificial intelligence.

^c^CTDI: computed tomography dose index.

^d^DLP: dose length product.

^e^CM: contrast medium.

### Risk-of-Bias Assessment

#### Overview

**Figure 2:**
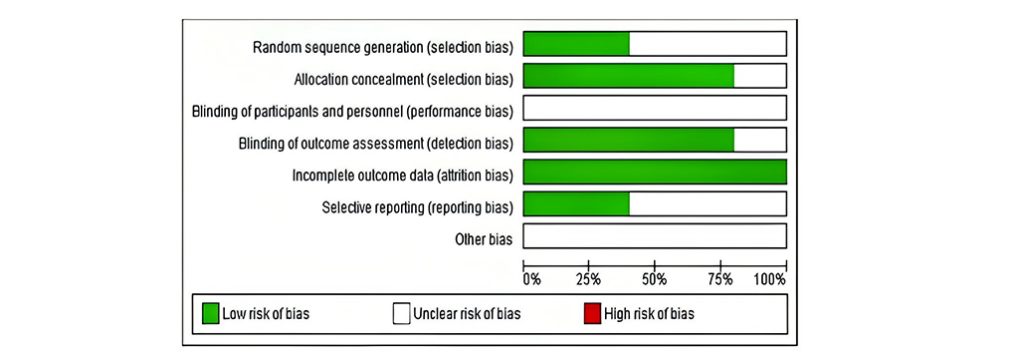
Risk-of-bias graph: review authors’ judgments about each risk-of-bias item presented as percentages across all included studies. This graph provides a visual representation of the risk of bias across different domains, indicating the methodological quality of the studies included in the meta-analysis.

The risk of bias for each included study was assessed using the Cochrane risk of bias tool. Figure 2 presents the risk of bias graph, showing the distribution of judgments across all included studies for each domain. Random sequence generation (selection bias) was assessed as having a low risk in 40% (2/5) of studies and unclear risk in 60% (3/5). Allocation concealment was judged as low risk in 80% (4/5) of studies and unclear in 20% (1/5). The blinding of participants and personnel (performance bias) was unanimously assessed as an unclear risk across all studies. Blinding outcome assessment (detection bias) was determined to be low risk in 80% (4/5) of studies and unclear in 20% (1/5). Incomplete outcome data (attrition bias) were consistently judged as low risk across all studies. Selective reporting (reporting bias) was assessed as low risk in 40% (2/5) of studies and unclear in 60% (3/5). Other bias was unanimously judged as unclear across all studies.

**Figure 3:**
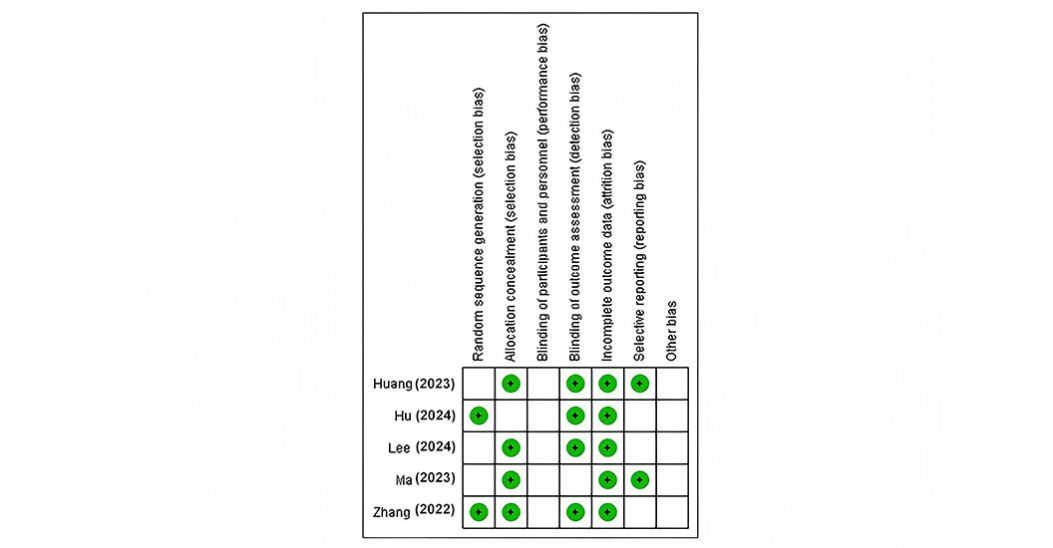
Risk-of-bias summary: review authors’ judgments about each risk-of-bias item for each included study. This summary offers a comprehensive overview of the risk of bias for each study, highlighting areas of strength and potential concerns within the meta-analysis.

Figure 3 [9,14-17] presents a comprehensive risk-of-bias summary for each study included in the analysis. Several key observations can be made from this summary. Notably, all 5 studies demonstrated a low risk for incomplete outcome data, indicating robust data collection and reporting practices. However, the blinding of participants and personnel was unclear across all studies, which is a common limitation in imaging studies where complete blinding can be challenging to achieve. Random sequence generation was assessed as low risk in 3 (60%) out of the 5 studies, suggesting generally sound randomization practices were used. Interestingly, the “other bias” category was consistently rated as unclear for all studies, pointing to potential unidentified sources of bias that may warrant further investigation. Finally, there was considerable variability in the assessment of selective reporting and blinding of outcome assessment across the studies, highlighting areas where methodological consistency could be improved in future research.

#### Image Quality

**Figure 4:**
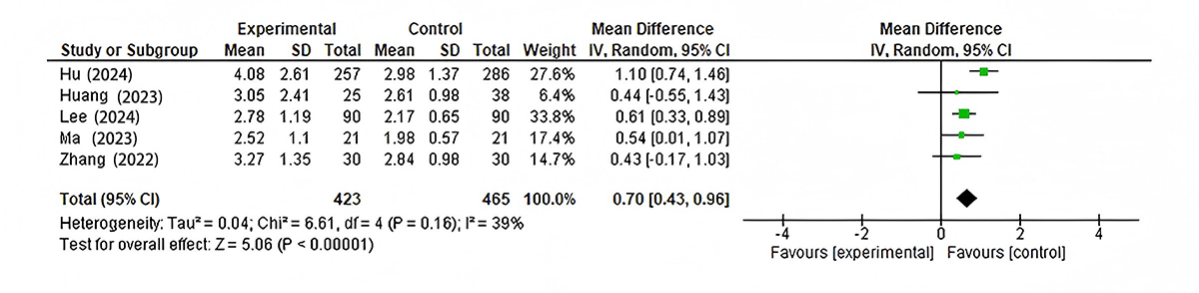
Forest plot comparing image quality scores between AI-based interventions and conventional CT imaging techniques across 5 clinical validation studies conducted from 2022 to 2024, focusing on various anatomical regions and clinical applications. This plot visually represents the mean differences in image quality, with confidence intervals, favoring either the experimental or control groups. AI: artificial intelligence; CT: computed tomography; IV: inverse variance.

All 5 studies reported on image quality outcomes. The meta-analysis showed a significant improvement in image quality with AI-based interventions compared to conventional methods (mean difference 0.70, 95% CI 0.43-0.96; *P*<.001; Figure 4 [9,14-17]). There was moderate heterogeneity among the studies (*I*²=39%), suggesting some variability in the effect of AI on image quality across different clinical contexts.

The largest effects were observed in the studies by Hu et al [14] and Lee et al [17], which focused on intracranial aneurysm detection and liver CT imaging, respectively. These studies carried the highest weights in the analysis (27.6% and 33.8%, respectively).

#### CT Dose Index

**Figure 5:**
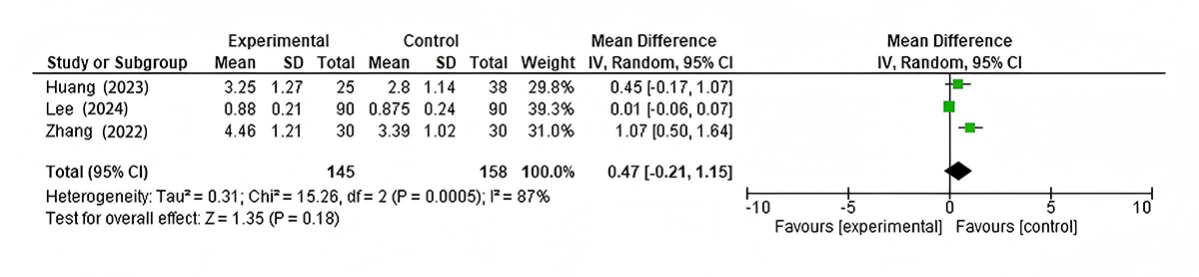
Forest plots of changes in CT dose index. This figure displays the impact of AI-based interventions on the CT dose index, with mean differences and CIs, across 3 studies that reported this outcome. AI: artificial intelligence; CT: computer tomography; IV: inverse variance.

Three studies [9,15,17] reported data on the CT dose index. The meta-analysis showed a trend toward reduction in CT dose index with AI-based interventions, but the result was not statistically significant (mean difference 0.47, 95% CI –0.21 to 1.15; *P*=.18; Figure 5 [9,14-17]). There was high heterogeneity among these studies (*I*²=87%), indicating substantial variability in the effect of AI on radiation dose across different applications.

The study by Zhang et al [15] showed the largest effect size in favor of AI, while Lee et al [17] reported almost no difference between the AI and conventional groups.

#### Efficiency in Image Analysis

**Figure 6:**
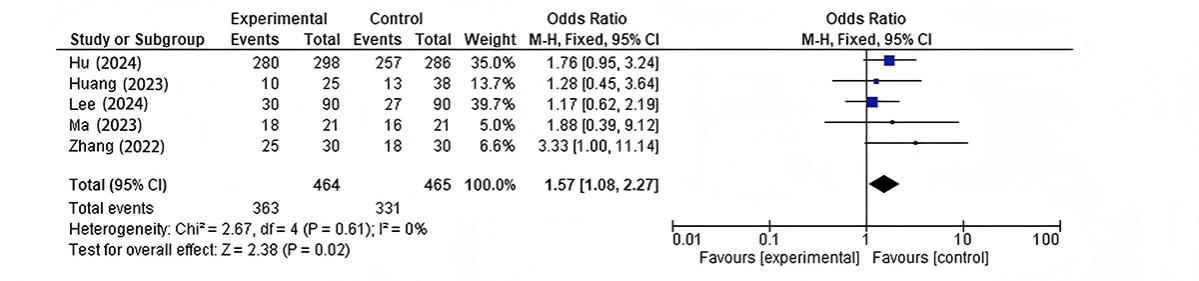
Forest plots of efficiency in image analysis. This forest plot demonstrates the comparison of image analysis efficiency between AI-based methods and conventional approaches, showing a significant improvement with AI across all 5 studies. AI: artificial intelligence; M-H: Mantel-Haenszel analysis.

All 5 studies provided data on the efficiency of AI in image analysis. The meta-analysis demonstrated a significant improvement in efficiency with AI-based methods compared to conventional approaches (odds ratio 1.57, 95% CI 1.08-2.27; *P*=.02; Figure 6 [9,14-17]). There was no significant heterogeneity among the studies for this outcome (*I*²=0%).

The study by Lee et al [17] had the largest weight (39.7%) in this analysis, likely due to its larger sample size. All studies showed an odds ratio favoring the AI group, although the precision of the estimates varied, with wider confidence intervals observed in smaller studies.

#### Diagnostic Performance

While not all studies reported on diagnostic performance metrics, those that did showed promising results. Hu et al [14] reported high accuracy (0.951), sensitivity (0.974), and specificity (0.928) for their AI model in detecting intracranial aneurysms. Lee et al [17] found that the diagnostic performance of low-dose CT with AI reconstruction was noninferior to standard-dose CT for liver lesion detection.

#### Publication Bias

**Figure 7:**
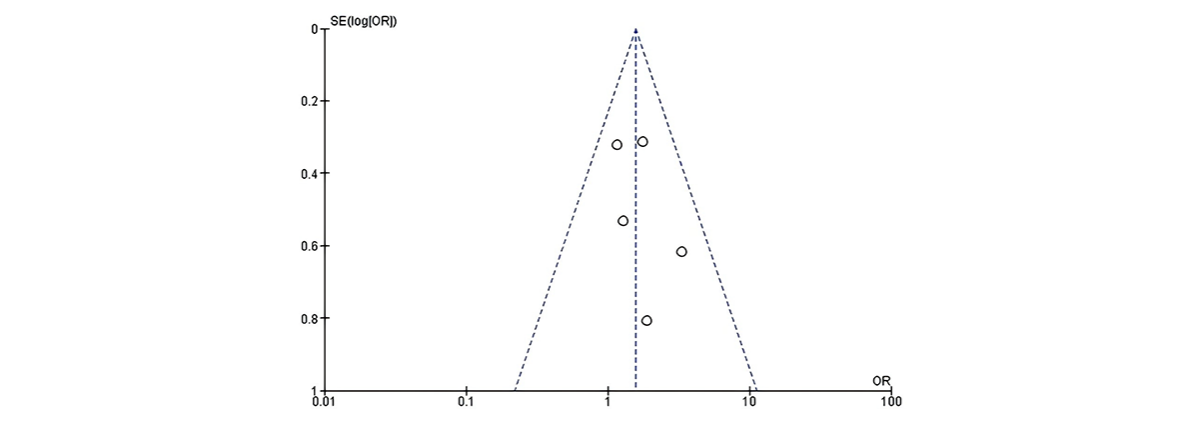
Funnel plot of efficiency in image analysis by AI. The funnel plot is used to assess publication bias, with the distribution of effect sizes visually represented against their precision. AI: artificial intelligence; OR: odds ratio.

Visual inspection of the funnel plot (Figure 7 [9,14-17]) did not reveal substantial asymmetry, suggesting a low risk of publication bias. However, the small number of included studies limits the reliability of this assessment.

## Discussion

### Principal Findings

This meta-analysis synthesized evidence from 5 clinical validation studies to evaluate the role of AI in CT image quality control and radiation protection. The findings suggest that AI-based interventions can significantly improve CT image quality and efficiency in image analysis, with a potential trend toward radiation dose reduction.

The significant improvement in image quality observed with AI-based interventions (mean difference 0.70, 95% CI 0.43-0.96; *P*<.001) is a key finding of this meta-analysis. This improvement was consistent across different anatomical regions and clinical applications, suggesting that AI algorithms have broad applicability in enhancing CT image quality. The enhanced image quality achieved through AI could have substantial clinical implications. Improved image clarity and detail may lead to more accurate diagnoses, potentially reducing the need for repeat scans or additional imaging studies [19]. This, in turn, could contribute to reduced overall radiation exposure for patients and improved workflow efficiency in radiology departments.

While the meta-analysis showed a trend toward CT dose index reduction with AI-based interventions, the result was not statistically significant (mean difference 0.47, 95% CI –0.21 to 1.15; *P*=.18). The lack of statistical significance does not necessarily imply that AI has no effect on radiation dose reduction. Rather, it highlights the need for further research to elucidate the factors influencing AI’s impact on radiation dose across different CT applications. It is possible that some AI algorithms are more effective at dose reduction than others, or that dose reduction capabilities may be more pronounced in certain anatomical regions or clinical scenarios [20].

Moreover, the trend toward dose reduction, even if not statistically significant, is encouraging. When combined with the significant improvements in image quality, these findings suggest that AI may enable a favorable shift in the balance between image quality and radiation dose in CT imaging [21].

The significant improvement in the efficiency of image analysis with AI-based methods (odds ratio 1.57, 95% CI 1.08-2.27; *P*=.02) is another important finding of this meta-analysis. This improvement in efficiency could have far-reaching implications for radiology workflows and patient care. Faster image analysis times could lead to reduced reporting turnaround times, potentially enabling quicker clinical decision-making and treatment initiation [22]. Additionally, improved efficiency could help address the growing demand for CT imaging services and the increasing workload on radiologists [23]. The lack of heterogeneity (*I*²=0%) in this analysis suggests that the efficiency gains with AI are consistent across different applications. This consistency is particularly noteworthy given the diversity of clinical scenarios represented in the included studies.

### Comparison With Prior Work

While not all studies reported comprehensive diagnostic performance metrics, those that did showed promising results. The high accuracy, sensitivity, and specificity reported by Hu et al [14] for intracranial aneurysm detection, as well as the noninferiority of low-dose CT with AI reconstruction for liver lesion detection reported by Lee et al [17], suggest that AI can maintain or even enhance diagnostic performance while potentially reducing radiation dose. These findings align with previous studies that have demonstrated the potential of AI to improve diagnostic accuracy in various imaging modalities [24]. The combination of improved image quality, potential dose reduction, and maintained or enhanced diagnostic performance positions AI as a powerful tool for advancing CT imaging practices.

The principle of action by which AI, particularly deep learning algorithms, enhances CT image quality is multifaceted. First, AI-driven approaches use advanced computational techniques to reconstruct images from raw data, optimizing the balance between image sharpness and noise reduction. This is achieved through the use of convolutional neural networks that learn to identify and enhance features of interest while suppressing background noise, leading to clearer and more detailed images [24]. Second, AI algorithms can adaptively adjust image reconstruction parameters based on the specific characteristics of the scanned tissue, resulting in improved contrast resolution and better visualization of anatomical structures. Third, AI-based techniques offer the capability for automated image analysis, which can standardize the interpretation process and reduce interobserver variability, thereby contributing to more accurate and consistent diagnostic outcomes [22]. These mechanisms collectively contribute to the significant improvement in image quality observed in our meta-analysis, underscoring the potential of AI to revolutionize CT imaging practices by enhancing diagnostic accuracy and patient safety.

### Strengths and Limitations

Several limitations of this meta-analysis should be noted. First, the small number of included studies limits the generalizability of the findings and precludes more detailed subgroup analyses. Second, the heterogeneity in AI algorithms, CT protocols, and outcome measures across studies made direct comparisons challenging. Third, the lack of long-term, follow-up data in the included studies means that the potential long-term impacts of AI-enhanced CT imaging on patient outcomes remain unclear. Future research should focus on larger, multicenter studies with standardized protocols and outcome measures to more definitively establish the impact of AI on CT image quality and radiation dose [25]. Long-term studies are needed to assess the clinical impact of AI-enhanced CT imaging on patient outcomes and radiation-induced cancer risk. Additionally, research into the cost-effectiveness of implementing AI in CT imaging workflows would be valuable for health care systems considering the adoption of these technologies.

### Conclusions

This meta-analysis provides evidence that AI-based interventions can significantly improve CT image quality and efficiency in image analysis, with the potential for radiation dose reduction. These findings suggest a promising role for AI in enhancing CT imaging practices, potentially leading to improved diagnostic accuracy, reduced radiation exposure, and enhanced patient care. As AI technologies continue to evolve, their integration into CT imaging workflows may become increasingly important in addressing the ongoing challenges of balancing image quality, radiation safety, and diagnostic accuracy.

## Appendix

Multimedia Appendix 1 Detailed Search Strategy for "The Role of Artificial Intelligence in CT Image Quality Control and Radiation Protection: A Meta-Analysis".

Multimedia Appendix 2 PRISMA checklist.

## Abbreviations

AI: artificial intelligence
CT: computed tomography
PRISMA: Preferred Reporting Items for Systematic Reviews and Meta-Analyses

## Data Availability

All data generated or analyzed during this study are included in this paper. Further inquiries can be directed to the corresponding author.
