# Supplementary material for: Effectiveness of AI for Enhancing Computed Tomography Image Quality and Radiation Protection in Radiology: Systematic Review and Meta-Analysis
Source: J Med Internet Res. 2025 Feb 27;27:e66622. doi: 10.2196/66622 (PMC11907168; doi:10.2196/66622)
Supplement: Multimedia Appendix 1 [file jmir_v27i1e66622_app1.docx]

Title: Detailed Search Strategy for "The Role of Artificial Intelligence in CT Image Quality Control and Radiation Protection: A Meta-Analysis"

1. Databases Searched:

PubMed

Embase

Web of Science

Science Direct

Cochrane Library

2. Search Time Frame:

The search was conducted without language restrictions and included studies published up to the search date in 2024.

3. Search Terms and Strings:

The search strategy employed a combination of Medical Subject Headings (MeSH) terms and keywords related to artificial intelligence, deep learning, and CT imaging. The specific search string used was:

("Artificial Intelligence"[Title/Abstract]) OR ("Computer Reasoning"[Title/Abstract]) OR ("Reasoning, Computer"[Title/Abstract]) OR

("AI"[Title/Abstract]) OR ("Machine Intelligence"[Title/Abstract]) OR ("Computational Intelligence"[Title/Abstract]) OR

("Computer Vision Systems"[Title/Abstract]) OR ("System, Computer Vision"[Title/Abstract]) OR

("Knowledge Acquisition, Computer"[Title/Abstract]) OR ("Knowledge Representations, Computer"[Title/Abstract]) OR

("Learning, Deep"[Title/Abstract]) OR ("Hierarchical Learning"[Title/Abstract]) AND

("CT Scan, X-Ray"[Title/Abstract]) OR ("CT X Ray"[Title/Abstract]) OR ("X-Ray Computer Assisted Tomography"[Title/Abstract]) OR

("Cine CT"[Title/Abstract]) OR ("Electron Beam Computed Tomography"[Title/Abstract]) OR

("Electron Beam Tomography"[Title/Abstract]) OR ("Tomography, X-Ray Computerized Axial"[Title/Abstract]) OR

("X-Ray Computerized Axial Tomography"[Title/Abstract])

4. Search Execution:

The search was conducted by two independent reviewers to ensure comprehensive coverage and reduce the potential for missed studies.

The search results were exported to a reference management software for de-duplication.

5. Records Identification:

Initial screening was based on titles and abstracts, followed by full-text review for potentially eligible studies.

Studies were included if they were clinical validation studies comparing AI-based interventions with conventional CT imaging techniques and reported outcomes related to image quality, radiation dose, or diagnostic performance.

6. Data Extraction and Quality Assessment:

Data extraction was performed using a standardized form that included study characteristics, sample size, patient demographics, AI intervention details, and outcome measures.

The quality of the included studies was assessed using the Newcastle-Ottawa Scale (NOS) for non-randomized studies.

7. Inclusion and Exclusion Criteria:

Inclusion criteria: Clinical validation studies comparing AI-based interventions with conventional CT imaging techniques; reported outcomes related to image quality, radiation dose, or diagnostic performance; and provided sufficient data for quantitative analysis.

Exclusion criteria: Non-clinical studies or those without a control group, studies focusing solely on AI development without clinical validation, review articles, case reports, conference abstracts, and studies with insufficient data for meta-analysis.

Methods: Summary of the Search Strategy

1. Search Strategy Summary:

In the Methods section, we will include a brief summary of our search strategy, mentioning the databases searched, the time frame of the search, and the key terms used to identify relevant studies.

2. Study Selection and Data Extraction:

We will summarize the process of study selection, including the number of records identified, screened, and included, as well as the rationale behind the inclusion and exclusion of studies based on the predefined criteria.

3. Quality Assessment:

A brief mention of the Newcastle-Ottawa Scale (NOS) used for assessing the quality of non-randomized studies will be included.
